# Starch dynamics as an important factor in early stages of somatic embryogenesis – a review

**DOI:** 10.3389/fpls.2026.1832558

**Published:** 2026-04-21

**Authors:** Miroslav Pernis, Jozef Mravec, Katarína Klubicová

**Affiliations:** Plant Science and Biodiversity Centre, Unit Institute of Plant Genetics and Biotechnology, Slovak Academy of Sciences, Nitra, Slovakia

**Keywords:** biochemical markers, embryogenic competence, induction, proliferation, starch

## Abstract

Somatic embryogenesis is an artificially induced process that results in the formation of an embryo without the fusion of gametes. Despite its significant potential for plant propagation and conservation of plant genetic resources, many unknowns remain regarding the specific mechanisms and the complexity of genetic, biochemical, and cellular regulations involved. The comparative data on induction of embryogenic tissue and the maturation of somatic embryos, along with additional results, suggest that complex carbohydrate dynamics are among the key factors involved. This review summarizes recent findings focused on early stages of SE, including induction, proliferation, comparison of embryogenic cell lines with contrasting embryogenic capacity, and loss of embryogenic capacity after prolonged cultivation obtained through various methods, including metabolomics, proteomics, transcriptomics, and cytological techniques/histochemical visualization, which highlight the regulation of the energy source starch. This encompasses its synthesis and remobilization, driven by related enzymatic machinery, as crucial for determining embryogenic capacity and prognosis in embryo maturation and germination in many economically important plant species. We also outline potential future directions, critical questions to address, and anticipated advancements in the topic, including the development of new research methodologies.

## Introduction

Somatic embryogenesis (SE) illustrates the remarkable ability of plant somatic cells to regenerate into complete plants through the formation of embryo-like structures, known as somatic embryos, under appropriate *in vitro* conditions. This propagation technology has been established for numerous plant species and represents an alternative vegetative propagation system for the rapid multiplication of plants with desirable traits (Zang et al., 2026; Fraga et al., 2023). When combined with cryopreservation, SE also contributes to the conservation of valuable plant genetic resources. Additionally, it provides an experimental model for studying developmental processes, such as cellular reprogramming and embryo patterning (Zimmerman, 1993; Peng et al., 2025).

Somatic embryos may develop directly from explant tissue (direct SE) or develop via an intermediate callus phase (indirect SE). During indirect SE, calli with varying embryogenic potential are generated, necessitating distinguishing between cell lines with different embryogenic potential and selecting the most productive ones. Early identification of highly productive cell lines is particularly important in species in which embryogenic capacity declines during long-term culture. Indirect SE typically proceeds through several steps: initiation, proliferation, pre-maturation, maturation, and somatic embryo germination (von Arnold et al., 2002). During induction, somatic cells acquire embryogenic competence, usually through the application of plant growth regulators such as auxins and cytokinins. Embryogenic tissues are subsequently multiplied during proliferation, while maturation occurs after transfer to media with modified hormone composition. Mature embryos germinate into plantlets under favorable conditions on a hormone-free medium (Hazubska-Przybył et al., 2016).

Despite successful protocols for many species, some drawbacks, such as low initiation frequency, culture growth and survival, loss of embryogenic capacity after prolonged cultivation, and low germination rates, need to be overcome to increase cost-effectiveness. Addressing these constraints is essential for improving the efficiency and cost-effectiveness of SE-based propagation systems (Guo et al., 2024).

Somatic embryogenesis involves extensive biochemical and physiological reprogramming regulated by stress signals and endogenous or exogenous plant growth regulators (Fehér, 2015; Asghar et al., 2023). Because embryogenic tissues differ substantially from non-embryogenic ones, considerable effort has been devoted to identifying reliable markers of embryogenic competence. These markers have been widely reviewed and include molecules of different biological levels, such as transcripts, transcription factors, proteins, metabolites, and cytological characteristics, which reflect the developmental state of embryogenic cultures (Rocha et al., 2016; Kurczyńska and Godel-Jędrychowska, 2023). Identifying practical markers is particularly important for the early selection of highly responsive cell lines and for monitoring developmental changes during SE. Among potential indicators, starch accumulation and distribution have attracted increasing attention, as they are easily visualized and quantified and may reflect metabolic changes associated with embryogenic competence. This review focuses on starch dynamics during early stages of SE, including induction, proliferation, comparison of embryogenic cell lines with contrasting embryogenic capacity, and loss of embryogenic capacity after prolonged cultivation (**Table 1**).

**Table 1:** List of selected publications describing the role of starch in early stages of somatic embryogenesis.

Plant group	Species	Methodology	Reference
Monocots	*Elaeis guineensis*, var. *Pisifera*	Histology (Lugol)	Almeida et al., 2020
	*Zea mays*	proteome	Liu et al., 2018
	*Brachypodium distachyon*	histology (the periodic acid-Schiff (PAS) reagent), *in situ* hybridization	Wehbi et al., 2022
	*Brachypodium distachyon*	histology (PAS)	Oliveira et al., 2017
	*Agapanthus praecox*	transcriptome	Yue et al., 2024
	*Saccharum* spp.	proteomic, histology?	Passamani et al., 2020
	Musa spp.	histology	Wang et al., 2013
Dicots	*Silybum marianum L.*	starch content; Anthrone-Sulfuric Acid	Abbasi et al., 2016
	*Pyrus communis*	starch content; Anthrone-Sulfuric Acid	Ameri et al., 2020
	*Medicago arborea*	starch content (amyloglucosidase)/aj iné sacharidy	Martin et al., 2000
Conifers	*Araucaria angustifolia*	the expression of target genes related to sucrose, raffinose, and starch metabolism; starch content (enzymatic determination)	Navarro et al., 2021
	*Pseudotsuga menziesii [Mirb.]*	histology Lugol (iodine potassium), biochemistry (enzymatic method), transcriptome, proteome, soluble sugars	Gautier et al., 2019
	*Pinus nigra*	secretome, extracellular α-amylase activity in liquid medium	Perniš et al., 2023
	*Pinus koraiensis*	starch content	Peng et al., 2022a
	*Pinus koraiensis*	transcriptome, proteome, starch content (Anthrone)?	Peng et al., 2022b
	*Cunninghamia lanceolata*	transriptome	Li et al., 2023
	*Pinus taeda*	starch content in the cells (sulfuric acid-anthrone method)	Silveira et al., 2004
	*Pinus koraiensis*	soluble sugars, starch content	Ren et al., 2022
	*Pinus halepensis*	histology, (light microscopy, TEM)	Pereira et al., 2020
	*Pinus Elliottii*	starch content (sulfuric acid-anthrone method)	Cheng et al., 2025

## Starch structure and function

Starch is a major carbon reserve synthesized in plastids, mainly in chloroplasts as transient starch and amyloplasts as storage starch, and is vital for plant metabolism and growth (Compart et al., 2021; Apriyanto et al., 2022). It consists of two glucose-linked polymers: amylose, a mostly linear polymer of α-1,4-linked D-glucose that forms helical structures, typically making up 15–30% of starch; and amylopectin, a highly branched polymer containing α-1,4 linkages with α-1,6 branch points every 20–25 glucose, which accounts for 70–85% of starch (Alcázar-Alay and Meireles, 2015).

These polymers form semi-crystalline granules, whose size and shape—such as spherical, oval, or polyhedral—differ among species and tissues. Crystalline regions, mainly formed by amylopectin double helices, alternate with amorphous zones rich in amylose, giving starch its characteristic birefringence under polarized light (Bertoft et al., 2024). Starch accumulates during active photosynthesis or when carbohydrate availability is abundant, and is degraded when energy demand increases or external carbon sources are scarce, releasing soluble sugars that support respiration and biosynthesis. These processes are tightly regulated by hormonal signals and developmental cues (Mijazawa et al., 1999). Starch granules can also function as statoliths, mediating gravitropism in plant organs (Leitz et al., 2009).

### Starch metabolism

**Figure 1:**
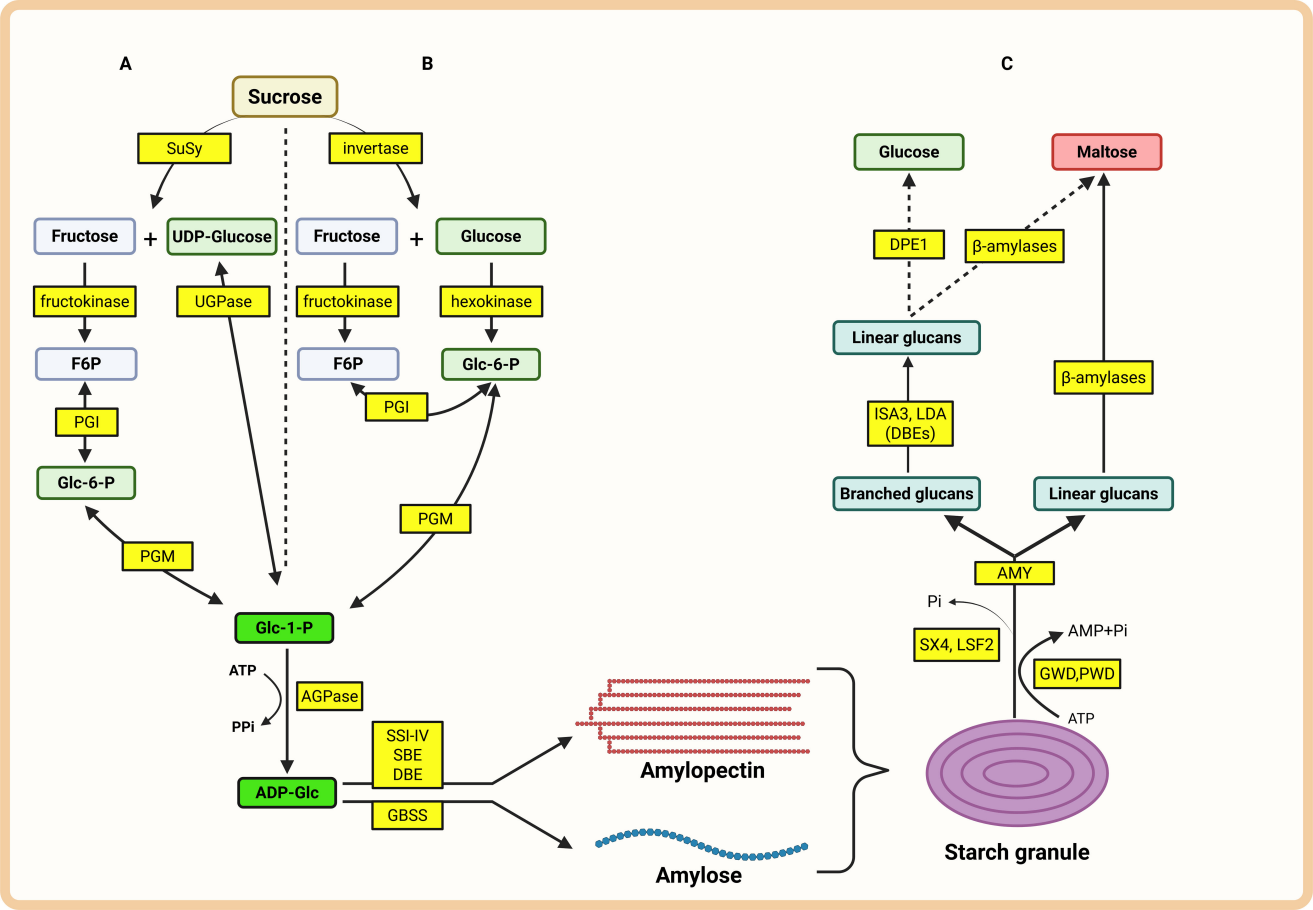
Simplified scheme of starch biosynthesis and degradation in heterotrophic tissue. Glucose-6-phosphate (Glc-6-P) for starch biosynthesis comes from sucrose. **(A)**: Sucrose synthase pathway. **(B)**: Invertase pathway. UDP-Glucose, uridin diphosphate glucose; UGPase, UDP-glucose pyrophosphorylase; F6P, fructose-6-phosphate; PGI, phosphoglucose isomerase; PGM, phosphoglucomutase; Glc-1-P, glucose-1-phosphate; PPi, inorganic pyrophosphate; AGPase, ADP-glucose pyrophosphorylase; ADP-Glc, ADP-Glucose; SSI-IV, soluble starch synthases; SBE, starch branching enzyme; DBE, starch debranching enzyme; GBSS, granule-bound starch synthase. **(C)**: starch degradation. GWD, glucan water dikinase; PWD, phosphoglucan water dikinase; SX4/LSF2, phosphoglucan phosphatases; Pi, inorganic phosphate; AMP, adenosine monophosphate; AMY, α-amylase; ISA3, isoamylase 3; LDA, limit dextrinase; DBEs, debranching enzymes; DPE1, disproportionating enzyme 1.

Starch metabolism involves various enzyme activities that drive synthesis, degradation, remodeling, and chemical modification, such as phosphorylation (Abt and Zeeman, 2020). In heterotrophic tissues, sucrose is converted into hexose phosphates via the sucrose synthase or invertase pathways, providing substrates for starch synthesis (**Figure 1**) (Navarro et al., 2021; Tetlow and Emes, 2017).

The rate-limiting step of starch biosynthesis is catalyzed by ADP-Glucose Pyrophosphorylase (AGPase), which converts glucose-1-phosphate into ADP-glucose, the main precursor of starch. Starch synthases elongate glucan chains, while branching enzymes introduce α-1,6 linkages to form amylopectin. Debranching enzymes remodel glucan chains to generate properly structured granules (Tetlow and Bertoft, 2020).

During starch mobilization, α-amylase cleaves internal α-1,4-glycosidic bonds, producing oligosaccharides such as maltose and maltotriose (Tetlow and Bertoft, 2020). β-amylase releases maltose from non-reducing ends of starch chains, while isoamylase and limit dextrinase hydrolyse α-1,6 linkages. The activity of these enzymes reflects different stages of starch turnover and can be combined with histological visualization of starch granules in explant tissues.

### Methodological approaches

Early investigations of starch dynamics during somatic embryogenesis relied primarily on histological localization, most commonly using Lugol staining, combined with measurements of starch content or starch-degrading enzyme activity (Godbole et al., 2004). Lugol’s solution (iodine-potassium iodide) stains amylose blue to blue-black and amylopectin reddish-brown, allowing starch granules to be visualized as dark oval or spherical structures (Zhao et al., 2023). The method is inexpensive and can be combined with other stains, such as Nile Blue, for lipid detection. The periodic acid–Schiff (PAS) reaction can also detect starch, but is less specific. Fluorescent visualization of starch can also be accomplished (Ovecka et al., 2012).

Starch quantification can be performed using several analytical approaches, each with distinct advantages and limitations. Colorimetric assays, such as the anthrone method, are simple and inexpensive, but measure total carbohydrates rather than starch specifically (Carillo et al., 2013). Therefore, ethanol pre-extraction is essential to remove soluble sugars and prevent significant overestimation. Enzymatic assays based on α-amylase and amyloglucosidase digestion, followed by glucose oxidase detection, offer higher specificity and accuracy but are more labor-intensive and costly. Gravimetric methods are also accurate but rarely used in routine analyses.

More recently, omics-based approaches have been applied as they do not directly measure/detect starch but instead enable elucidation of the mechanisms underlying starch metabolism during SE (Li et al., 2023). Transcriptomic, proteomic, and metabolomic analyses enable the identification of genes and pathways associated with starch metabolism and embryogenic competence. Although these techniques provide comprehensive insights, their high cost often limits analyses to a small number of genotypes. Candidate markers identified through omics must therefore be validated across broader genetic backgrounds and through functional studies (Gulzar et al., 2020; Kurczyńska and Godel-Jędrychowska, 2023).

Because early SE stages involve extensive metabolic reprogramming, combining histological, biochemical, and molecular approaches provides the most comprehensive framework for investigating starch dynamics during the induction and proliferation phases.

### Starch and embryogenic potential

Cell lines with high embryogenic capacity produce a large number of high-quality somatic embryos and are often referred to as responsive cell lines, in contrast to those with low embryogenic capacity or blocked cell lines (Peng et al., 2022a). Reliable evaluation of the embryogenic capacity of induced cell lines is therefore crucial for the effectiveness of the entire process. However, in most systems, this evaluation is only possible after a maturation test, which is time-consuming. The ability to distinguish cell lines with contrasting embryogenic potential at a very early stage would be beneficial, particularly in conifers, where the loss of embryogenic capacity after long-term cultivation is a major limitation.

Starch accumulation patterns differ markedly between embryogenic and non-embryogenic tissues, although the observed trends vary among plant species. In the embryogenic tissue of *Pseudotsuga menzii*, starch grains were scarce and localized mainly in the distal part of the suspensor. In contrast, non-embryogenic callus (NEC) contained starch in almost all cells, resulting in significantly higher overall starch concentrations (Gautier et al., 2019). A similar pattern was observed in *Medicago arborea*, where higher levels of starch also accumulated in NEC (Martin et al., 2000). Likewise, higher starch content was observed in blocked cell lines compared with responsive cell lines of *Pinus koraiensis* (Peng et al., 2022a), and a negative relationship between embryogenic competence frequency and starch content was also reported in *Pyrus communis* (Ameri et al., 2020).

In contrast, several studies have reported the opposite trend. In dicotyledonary species *Silybum marianum* L., embryogenic tissue contained significantly higher starch levels than NEC (Abbasi et al., 2016). Similarly, histological observations in monocot *Elaeis guineensis*, var. Pisifera revealed a greater accumulation of starch granules in embryogenic cells (Almeida et al., 2020). Comparable results were obtained in Musa spp., where embryogenic cells contained larger and more abundant starch grains than non-embryogenic cells (Wang et al., 2013).

However, not all studies detected differences in starch accumulation during early developmental stages. For example, in *Araucaria angustifolia*, no significant difference in starch content was observed between lines with contrasting responsiveness at the proliferation stage (Navarro et al., 2021). Recent studies employing omics approaches provide additional support for the theory that sucrose and starch metabolism play a role in somatic embryogenesis (Wang et al., 2021; Yue et al., 2022). Comparative proteomic analysis revealed that enzymes involved in sucrose and starch degradation accumulated in EC, and conversely, enzymes active in synthesis accumulated in NEC (Liu et al., 2018). Similarly, the comparative secretome (extracellular proteome) analysis of *Pinus nigra* embryogenic tissue with low and high embryogenic capacity revealed that α-amylase accumulation and activity were higher in cell lines with high embryogenic capacity (Perniš et al., 2023).

Long-term subculture represents another factor influencing starch metabolism and embryogenic competence. During extended cultivation, embryogenic tissues undergo changes in physiology and biochemistry, leading to declines or loss of the ability to produce somatic embryos. Changes in protein accumulation associated with this loss have been shown to be genotype-specific (Klubicová et al., 2017). Transcriptomic analysis revealed variability in starch metabolism between responsive and blocked cell lines and in tissues that had lost embryogenic capacity. Genes encoding granule-bound starch synthase, which catalyzes starch synthesis, were highly expressed in blocked cell lines. In tissues after loss of embryogenic capacity, genes encoding 1,4-α-glucan branching enzyme were highly expressed. This enzyme increases the degree of starch branching and slows its degradation, suggesting that starch structure and branching patterns may influence embryogenic competence in addition to total starch content. Differences were also observed in the expression of starch-degrading enzymes, with α-amylase and β-fructofuranosidase genes highly expressed in responsive and blocked cell lines, whereas most β-amylases were highly expressed in cell lines after loss of embryogenic capacity (Peng et al., 2022b).

Consistent with these observations, starch content decreased during the prolonged subculture of *Pinus Eliottii* embryogenic tissue. The highest starch level was observed in early subcultures, whereas later passages showed a sharp decline in starch content, accompanied by reduced embryogenic competence (Cheng et al., 2025). Culture conditions can also influence both starch metabolism and embryogenic potential. The length of the subculture cycle affected the ability of embryogenic tissue to produce somatic embryos and associated biochemical parameters, including starch content. In *Pinus massoniana*, a seven-day subculture interval maintained embryogenic competence longer than a fourteen-day interval and resulted in higher starch levels in the tissue (Ren et al., 2022).

Plant growth regulators also affect starch dynamics and embryogenic competence. Auxin and cytokinins have opposite effects on amyloplast development and the expression of starch synthesis genes (Miyazawa et al., 1999). In some systems, embryogenic callus cultured without 2,4-D maintained higher embryogenic competence and contained more starch granules than callus maintained on auxin-containing medium. Interestingly, increased accumulation of the starch-degrading enzyme α-amylase was associated with the maintenance of embryogenic competence, despite higher starch content and larger starch granules in tissues cultured without 2,4-D (Passamani et al., 2020).

Other physiological factors may also influence starch metabolism during somatic embryogenesis. Nitric oxide affected starch content differently in cell lines with contrasting embryogenic responsiveness in *Araucaria angustifolia*, suggesting that modulation of starch accumulation may help optimize *in vitro* conditions for somatic embryo development (Navarro et al., 2021). Temperature can also influence starch accumulation and embryogenic development. Pereira et al. (2020) observed high starch accumulation in embryogenic calli under different temperature treatments, while elevated temperature promoted higher somatic embryo production during maturation.

Overall, the available evidence indicates that starch accumulation patterns differ among species, developmental stages, and culture conditions. Consequently, starch cannot be considered a universal marker of embryogenic competence. Nevertheless, in certain species, starch metabolism and its spatial distribution may serve as useful indicators for the early identification of tissue with embryogenic potential.

## Discussion and future perspective

Studies conducted over several decades across a wide range of plant species have demonstrated that starch serves as a dynamic, tightly regulated carbon reserve during somatic embryogenesis. Its accumulation and timely mobilization provide energy and carbon skeletons required for cellular reprogramming, cell division, and early embryo development. Observations at the cellular level further support a link between starch metabolism and cell proliferation. For instance, in Brachypodium, actively dividing cells typically lacked densely packed starch granules, while cells completely devoid of starch reserves did not divide, suggesting that a minimal carbohydrate reserve may be required to sustain cell division during early embryogenic development (Wehbi et al., 2022).

In many systems, the spatial distribution of starch granules within embryogenic tissues has also been proposed as a practical histological indicator of embryogenic competence. However, available evidence indicates that both the localization and quantity of starch are highly species-dependent and strongly influenced by culture conditions, such as carbon source, osmotic environment, and plant growth regulators (Hazubska-Przybył et al., 2016; Yue et al., 2024).

Recent advances in transcriptomic, proteomic, and metabolomic analyses have reinforced the view that starch metabolism is closely integrated with central energy metabolism and developmental regulatory networks during somatic embryogenesis (Zhang et al., 2019; Chen et al., 2023). Changes in the expression of genes encoding enzymes involved in starch synthesis and degradation frequently accompany the acquisition or loss of embryogenic competence. Consequently, starch metabolism may represent both a physiological readout of embryogenic status and a potential target for optimizing somatic embryogenesis protocols (Zdravković-Korać et al., 2026).

Despite these advances, several important questions remain unresolved. The mechanisms linking starch metabolism to signaling pathways that regulate embryogenic competence remain poorly understood. In particular, the relationship between starch metabolism and other carbohydrate-related processes, such as cell wall polysaccharide biosynthesis and remodeling, remains largely unexplored. Furthermore, starch may have additional roles beyond functioning as a simple storage compound. Recent observations describing amyloplast autophagy suggest that starch-containing plastids may participate in cellular recycling pathways during embryogenic development (Gao et al., 2025), highlighting new directions for future research.

Another unresolved aspect concerns the mechanism of starch remobilization in embryogenic cultures. Elevated accumulation and activity of extracellular α-amylases detected in the secretome of highly embryogenic pine tissues may indicate enhanced carbohydrate recycling from surrounding cells or tissues undergoing programmed cell death (Perniš et al., 2023). Programmed cell death occurs in two waves during conifer somatic embryogenesis: the first during the transition from proembryogenic masses to developing embryos, and the second during the elimination of terminally differentiated suspensor cells (Filonova et al., 2000). In this context, extracellular α-amylases may redistribute carbon resources to support rapidly developing embryogenic cells. However, the precise origin and function of these enzymes remain unclear. It is also possible that their accumulation reflects stress-related responses rather than a direct role in starch degradation (Doyle et al., 2007). Therefore, α-amylase activity may represent a physiological marker of embryogenic competence rather than a direct regulatory factor.

Although omics technologies have provided valuable insights into the molecular basis of somatic embryogenesis, functional validation of candidate genes remains limited. Future research should therefore focus on genetic approaches capable of directly testing the role of starch metabolism in embryogenic competence. Genome-editing technologies such as CRISPR/Cas9 offer promising opportunities to investigate the functions of genes involved in starch biosynthesis and degradation in both model and non-model plant species (Ghosh, 2024; Deligani et al., 2026). Such approaches have already been successfully applied to modify starch metabolism in several crops, including potato (Johansen et al., 2019). Targeted manipulation of starch-related genes through gene knockdown, overexpression, or pharmacological modulation may help clarify the role of starch in regulating developmental processes during somatic embryogenesis.

In addition to genetic approaches, methodological advances in starch analysis are opening new possibilities for studying starch dynamics in embryogenic tissues. Modern *in situ* imaging techniques, such as the NegFluo method, combine confocal microscopy with computational image analysis to visualize and quantify starch granules in tissues non-destructively (Vandromme et al., 2019). Highly sensitive spectrophotometric assays have also been developed to detect very small quantities of starch, which is particularly useful when working with small or early-stage embryogenic samples. Furthermore, advanced analytical techniques such as nuclear magnetic resonance (NMR), Fourier-transform infrared spectroscopy (FTIR), X-ray diffraction, and atomic force microscopy provide detailed information on starch structure, crystallinity, and granule organization.

The integration of these techniques with omics approaches represents a promising direction for future research. Combining imaging, spectroscopy, and molecular analyses may enable the development of multi-scale models linking starch distribution, metabolism, and gene regulation during somatic embryogenesis. Such integrative approaches could significantly improve our understanding of the metabolic and developmental processes underlying embryogenic competence. Further progress may also benefit from the application of carbohydrate-specific analytical tools. For example, the use of anti-amylose antibodies such as INCH1 (Rydahl et al., 2017) could facilitate more precise visualization of starch granules and their spatial organization in embryogenic tissues. Complementary approaches, such as glycomic profiling (e.g., CoMPP or MAPP), may provide comprehensive information on the composition and dynamics of complex carbohydrates in developing cultures. Emerging techniques, such as single-cell transcriptomics, also hold great promise for resolving cell-type-specific metabolic processes during the early stages of somatic embryogenesis (Zheng et al., 2023).

Ultimately, a deeper understanding of starch metabolism and its relationship with embryogenic competence will be essential for improving the efficiency and reproducibility of somatic embryogenesis protocols. Identifying reliable biochemical markers, including those associated with starch metabolism, may facilitate the early selection of highly responsive cell lines and enhance the application of somatic embryogenesis in modern plant biotechnology.
